# The canine vaginal microbiome during heat and fertility in healthy breeding dogs

**DOI:** 10.1371/journal.pone.0321683

**Published:** 2025-04-28

**Authors:** Anna Sophia Leps, Eva-Maria Packeiser, Christina Schwens, Benjamin Stoelcker, Semir Doric, Martina Wirkner, Beate Walter, Axel Wehrend, Viktoria Kichmann, Klaus Jung, Sandra Goericke-Pesch

**Affiliations:** 1 Unit for Reproductive Medicine – Clinic for Small Animals, University of Veterinary Medicine Hannover, Foundation, Hannover, Germany; 2 Antech Lab Germany GmbH, Augsburg, Germany; 3 SYNLAB MVZ Weiden GmbH, Dept. of Molecular Biology, Weiden, Germany; 4 Clinic of Small Animal Surgery and Reproduction at the Centre for Clinical Veterinary Medicine, Faculty of Veterinary Medicine, LMU Munich, Germany; 5 Veterinary Clinic for Reproductive Medicine and Neonatology, Faculty of Veterinary Medicine, Justus-Liebig-University, Giessen, Germany; 6 Institute for Animal Genomics, University of Veterinary Medicine Hannover, Foundation, Hannover, Germany; University of Life Sciences in Lublin, POLAND

## Abstract

A healthy and balanced vaginal microbiome is often thought to be an important prerequisite for successful breeding and healthy litters. Previous studies investigating the influence of canine vaginal bacteria on fertility mostly relied on aerobic culturing. In recent years, culture-independent methods, such as next-generation sequencing (NGS), have become popular. With the ability to analyze the microbiome as a whole, research in this field has made notable advances. This is the first study to correlate NGS data of the canine vaginal microbiome in heat with fertility data. Healthy breeding bitches (n=80) presented for routine pre-breeding examination were sampled during early heat and mated after ovulation. A vaginal sample was taken for NGS analysis and microbiological culture. Additionally, a blood sample was collected. Fertility data (mating, pregnancy, delivery, litter size) were assessed. NGS revealed a diverse microbiome in all the samples. Bioinformatics and statistical analysis did not provide evidence of larger differences in the microbiome of those bitches that became pregnant and those that did not.

## Introduction

The canine vaginal microbiome composition and its impact on fertility is of particular interest for breeders and veterinary professionals. It has been described previously by numerous authors, most using aerobic culturing [1–10]. In recent years, there has been a notable increase in the use of molecular, culture-independent methods, such as next-generation sequencing (NGS), as these offer a valuable insight into the complete microbiome within a specimen [11]. A variety of microbial ecosystems have been identified within the organs of dogs [12–17]. Furthermore, additional microbial ecosystems have been identified, including the reproductive microbiome in cattle, sheep, pigs, and rabbits [18–24]. The vaginal [25,26], uterine [25,27] and semen microbiota [28] of the dog have all been described previously in various studies. Despite the impact of the stage of the estrus cycle [25] and the age (prepubertal/adult) [26], a description of the canine vaginal microbiome in relation to fertility of healthy breeding dogs is still missing.

When considering conventional aerobic culture, differentiation between physiological and unphysiological flora is challenging, yet bacteria are often regarded as a causal factor of infertility and abortion in small animals [29–31]. This is mainly because most bacteria, except for *Brucella canis* [30, 31], act as opportunistic pathogens. Some species are frequently found in the context of reproductive tract disease, like *Escherichia coli* in dogs suffering from pyometra [32,33], or puppy death [32]. Beta-hemolytic streptococci and *Staphylococcus intermedius* are further species often related to reproductive tract disease [32].The aforementioned species are also frequently observed in non-diseased bitches [3–5,9,10,34]. There is some evidence to suggest that their occurrence in pure culture or a mixed culture with only a few isolates and a higher quantity of bacteria is more often correlated with reproductive tract disease [32,34]. It has to be considered that many factors such as cycle stage [4,5,8,10] but also sampling location [3,34] have an influence on the bacterial composition. However, a decision as to whether a bitch has a reproductive pathology or not cannot solely be made by identifying specific isolates in aerobic culture, thus emphasizing the need for a thorough gynecological examination.

The influence of *Mycoplasma* spp. on fertility is subject of discussion [35]. Although there is evidence that *Mycoplasma* spp. are frequently found in healthy bitches [10,36], many breeders and also veterinarians fear subfertility and infertility of bitches as well as stud dogs due to *Mycoplasma* spp.. Due to the lack of a rigid cell wall, *Mycoplasma* spp. are intrinsically resistant against antimicrobials targeting the bacterial cell wall, such as β-Lactam antibiotics [30,37]. Besides, they are usually not identified with standard bacteriological culture, but require special media and time- and labor-intensive culturing methods [37]. For this reason, many commercial laboratories do not offer *Mycoplasma* culturing, but identification on the genus level by PCR [37]. As a consequence of breeders’ fear of *Mycoplasma* spp., clinically healthy breeding dogs are routinely tested and, unfortunately, in case of a positive PCR result treated with antimicrobials, such as tetracyclines, mostly doxycycline, or fluoroquinolones [30,37,38]. However, a general *Mycoplasma* spp. PCR is not useful, as only certain species, such as *Mycoplasma canis* [38,39], *Mycoplasma spumans*, and *Mycoplasma maculosum* [40], have been related to reproductive tract disease in the bitch [38,39]. *Ureaplasma* spp. were suggested to be possibly related to infertility/reproductive-tract disease in one study, but without conclusive evidence [36]. Consequently, identification at species level may be useful to distinguish commensal from potentially pathogenic *Mycoplasma* spp.. Additionally, more evidence of the influence of certain species on fertility and reproductive health is needed to avoid unnecessary antimicrobial use.

Since evidence of the relation between vaginal microbiota, including *Mycoplasma*, and fertility is conflicting, more research is needed to limit antimicrobial treatment to cases with a clear indication. This is crucial regarding the threat of increasing antimicrobial resistance in both human and veterinary medicine [41]. Furthermore, close contact with companion animals poses a risk for transmission of resistance genes [42]. Therefore, prudent use of antimicrobials in pet animals is also of importance for human patients to reduce the selective pressure [43].

Due to the lack of evidence of a possible correlation of vaginal microbiota with fertility in the dog, the aim of the present study was to gain knowledge on the composition of the vaginal microbiota of fertile and non-fertile bitches (represented by clinically healthy breeding bitches becoming pregnant or not after successful mating/insemination), as analyzed by NGS. This would possibly allow further conclusions to be drawn regarding the richness and diversity of microbiota as well as the presence of *Mycoplasma* for reproductive performance.

## Materials and methods

### Animals and experimental design

Bitches included in the study were presented for pre-breeding examination including microbiological sampling between March 2022 and October 2023 with the owner’s consent. The sampling protocol was approved by the Animal Welfare Officer of the University of Veterinary Medicine Hannover, Foundation, Hannover, Germany. The animals were presented to one of the listed facilities: Unit for Reproductive Medicine – Clinic for Small Animals, University of Veterinary Medicine Hannover, Foundation (n=72), Veterinary Clinic for Reproductive Medicine and Neonatology, Faculty of Veterinary Medicine, Justus Liebig University Giessen, Giessen, Germany (n=19), and Clinic of Small Animal Surgery and Reproduction at the Centre for Clinical Veterinary Medicine, Faculty of Veterinary Medicine, Ludwig Maximilian University of Munich, Munich, Germany (n=5).

In total, 96 bitches were included in the study. All bitches were clinically healthy, adult breeding dogs. They underwent a physical examination and subsequent gynecological examinations, including vaginoscopy. Multiple samples, including microbiological specimens, swabs for vaginal cytology and a blood sample, were collected.

If feasible, follow-up examinations were undertaken until ovulation. Subsequently, the bitches were either mated naturally or artificially inseminated. Pregnancy diagnosis using ultrasound was performed in all bitches. Owners were contacted for a follow-up phone call after the expected delivery for information about antimicrobial treatment during the pre-breeding period or after breeding for breeding outcome (pregnancy yes/no, delivery yes/no) and litter size.

### Sample collection

At the first pre-breeding consultation, two microbiological swabs were collected during heat before mating using a sterile tube speculum (Model “Hannover”, Wirtschaftsgenossenschaft Deutscher Tierärzte eG, Garbsen, Germany). To avoid contamination, any hair in the perineal region of the bitches was held aside by an assistant and the vulva was cleaned with a dry towel before inserting the sterile tube speculum. All samples were taken from the cranial vaginal mucosa using sterile cotton swabs. One swab was transferred to a sterile amies medium with charcoal (Transsystem Sterile Transport Swab, COPAN Italia, Brescia, Italy) for aerobic and anaerobic culturing and the other one to a DNA/RNA Shield™Collection Tube w/ Swab (ZymoResearch Corp., Irvine, CA, USA) for NGS analysis under sterile conditions. The swabs were transported to the laboratory on the same day via courier. A swab for vaginal cytology was then obtained using a Kilian speculum (Wirtschaftsgenossenschaft Deutscher Tierärzte eG, Garbsen, Germany) to evaluate the cycle stage of the bitches. A sterile cotton swab was moistened with a sterile sodium chloride solution and cell material was collected in a rolling motion over the mucosal surface of the dorsal vaginal wall. The obtained cell material was directly rolled onto a microscopic slide (various manufacturers) and air-dried. Once dried, the slides were stained using a rapid staining solution after the manufacturer’s instructions (Hemacolor Rapid staining of blood smear, Merck KGaA, Darmstadt, Germany). Vaginal cytology was evaluated using light microscopy.

### Sequencing and data analysis

Samples for NGS analysis were stored at 5–25 °C in a DNA/RNA Shield™Collection Tube w/ Swab (ZymoResearch Corp., Irvine, CA, USA) before shipping to the SYNLAB MVZ Weiden GmbH, Department for Molecular Biology, NGS Laboratory, Weiden, Germany. Once received, samples were spiked with the ZymoBIOMICS™ Spike-in Control I (High Microbial Load) in accordance with the manufacturer’s instructions (ZymoResearch Corp., Irvine, CA, USA) prior to extraction. The ZymoBIOMICS® Microbial Community DNA Standard II was employed as the positive control, the DNA/RNA shield medium was used as the negative control. DNA extraction was performed using the Chemagic™ Viral DNA/RNA 300 DNA Isolation Kit (Revvity Cellular Technologies GmbH, Hamburg, Germany). The average DNA yield after extraction was 0.9 ng/µl with a 260/280 ratio between 1.8 and 2.0, indicating a high purity. Amplicon libraries were sequenced in duplicates for each sample with primers targeting the V3-V4 region using the 16S Metagenomic Sequencing Library Preparation protocol (Illumina Inc., San Diego, CA, USA). DNA concentration was measured for all samples using the Fluoroskan (Thermofisher Scientific) and normalised to 4ng/µl after library preparation. Subsequently, 300-base paired-end reads were generated on the Illumina MiSeq platform (Illumina Inc.) Sequencing data analysis were performed with the Illumina Basespace 16S Metagenomics app v1.1.2. using the RefSeq RDP 16S v3 May 2018 DADA2 pipeline for species-level taxonomy profiling (Illumina Inc.), yielding raw read counts. All samples were reprocessed uniformly in silico, removing the Spike-in Control I microbial abundance from raw data. Sensitivity threshold was set to 0.01% relative abundance. Diversity indices were calculated with mean relative abundance values from the duplicate measurements.

### Blood sampling and analysis

Blood samples were drawn from all bitches repeatedly for progesterone monitoring to determine the ovulation indirectly. Concomitant with one sampling for progesterone, blood samples for hematology, blood chemistry analysis, and total thyroxine (T4) measurement were collected. Serum tubes (Wirtschaftsgenossenschaft deutscher Tierärzte eG) were used for progesterone analysis, EDTA tubes (BD Vacutainer EDTA tube, Becton Dickinson GmbH, Heidelberg, Germany) for hematology and serum separator tubes (BD Vacutainer SST, Becton Dickinson GmbH) for blood chemistry analysis and T4 measurement.

The blood samples drawn for progesterone monitoring were analyzed on the same day with an enzyme-linked fluorescent assay using Mini VIDAS (bioMérieux Deutschland GmbH, Nürtingen, Germany).

Blood samples for hematology, blood chemistry, and T4 measurement were processed and sent to the laboratory (Antech Lab Germany GmbH, Augsburg, Germany) on the same day. Serum separator tubes were centrifuged after coagulation time and serum was transferred to a plain tube. The plain tubes containing serum and EDTA tubes were then sent to the analyzing laboratory for further analysis.

Hematology samples were analyzed using flow cytometry on ADVIA ® 2120i (Siemens Healthineers AG, Forchheim, Germany). AU5822 (Beckman Coulter GmbH, Krefeld, Germany) was used to assess blood chemistry photometrically and electrolytes (sodium, chloride, potassium) with an ion-selective electrode. T4 measurements were carried out using a chemiluminescent immunoassay (Immulite ® 2000 xPi, Siemens Healthineers AG).

### Statistical and bioinformatics data analysis

All analyses were performed using the programming environment R (V 3.4.4, www.r-project.org) as well as packages from the Bioconductor repository (www.bioconductor.org).

Animal characteristics were compared between pregnant and non-pregnant individuals using either t-tests or the Fisher’s exact test. The significance level for these tests was set to α=5%. Characteristics were described using mean +/- standard deviation or absolute and relative frequencies.

Raw sequencing read counts were transformed into metric continuous data using the voom algorithm combined with quantile normalization [44]. Normalized microbiome profiles were visualized using clustered heatmaps and principal component plots. Differential species analysis was performed using the functionality of the limma-package [45] where normalized abundances were modelled with pregnancy-status as explanatory variable. We ran a second model to study the effect of potentially confounding variables. Raw, species-specific *p* values were adjusted by the method of Benjamini and Hochberg [46] to control a false discovery rate (FDR) of 5%. *P* values and log2 fold changes were visualized by volcano plots.

Alpha diversity was quantified by different species richness scores (Observed, Chao1, ACE, Shannon, Simpson, Fisher) implemented in the estimate richness function of the phyloseq package [47]) and compared between pregnant and non-pregnant individuals by the Wilcoxon rank sum test. Again, a significance level of α=5% was used.

Microbiome analyses were first performed at phylum level as described above, then also at genus level, and for *Mycoplasma* at species level.

## Results

### Animals, grouping, antimicrobial treatment

Physical and gynecological examination did not reveal any abnormalities, including abnormal vaginal discharge or signs of inflammation in vaginal cytology in any of the bitches. Of all 96 bitches, 16 had to be excluded due to not being mated for personal reasons of the owners (n=13), due to a suspected ovarian pathology diagnosed at pregnancy ultrasound (n=1) or a lack of follow-up with the owner for pregnancy outcome (n=2). The remaining animals were assigned to the pregnant group (n=61) or non-pregnant group (n=19).

The remaining dogs included in the study (n=80) belonged to 37 different breeds and one bitch was a mixed breed. They had a mean of 4.0 ± 1.7 years old (min.-max.: 1.4–8 years) and had a mean body weight of 30.1 ± 14.2 kg (min.-max.: 4.9 kg – 71.3 kg). The non-pregnant dogs (n=19) (4.9± 1.7 years, min.-max.:1.4–8 years) were significantly older compared to the pregnant dogs (n=61) (3.8 ± 1.6 years, 1.4–7.0 years) (*p*=0.01) (Table 1). The number of puppies ranged from 1 to 15 puppies per bitch (mean: 6.9 ± 3.0). In total, 419 puppies were born to 61 bitches.

**Table 1:** Comparison of clinical variables between pregnant (n=61) and non-pregnant (n=19) healthy breeding bitches. Results are presented either as mean ± standard deviation or absolute (relative) frequencies. *P* value indicates the result of statistical analysis; *p* <0.05 is considered statistically significant.

	Pregnant	Non-pregnant	*p*
**Age (years)**	3.8±1.6	4.9±1.7	0.01
**Weight (kg)**	28.9±15.1	34.0±10.3	0.10
**Day of sampling in relation to day of heat**	4.5±1.9	5.1±2.1	0.36
**Antimicrobial treatment after sampling**			0.03
No	57 (93%)	14 (73%)	
Yes	4 (7%)	5 (26%)	
**Type of mating**			1.0
Natural mating	47 (82%)	15 (83%)	
Artificial insemination	10 (18%)	3 (17%)	
No information available	4	1	
**Proof of ovulation before mating**			0.41
Yes	42 (69%)	11 (58%)	
No/ No information available	19 (31%)	8 (42%)	

Of all included animals (n=80), nine dogs received antimicrobial treatment after sampling and before mating/insemination, with either amoxicillin-clavulanic acid (n=5), trimethoprim-sulfamethoxazole (n=3), or enrofloxacin (n=1). In all cases, antibiotic treatment was started due to the results of the aerobic culture and/or history of reproductive failure. Group comparison revealed significantly fewer treated animals in the pregnant (n=4; 7%) than in the non-pregnant group (n=5; 26%) (*p*=0.03).

The type of mating was described for 75 dogs, with 62 bitches being mated naturally and 13 bitches being artificially inseminated with either fresh, chilled, or frozen semen. For the remaining 5 bitches, no information was given by the owners concerning the type of mating. Composition of groups did not differ statistically. Regarding the correct timing of the mating, proof of ovulation was obtained in 53 dogs (pregnant: n=42, non-pregnant n=11). For the remaining dogs either no proof of ovulation was obtained, or no reliable information was available. Statistical analysis revealed no significance (Table 1).

### Blood analysis

Results from the blood analysis were available for 78 dogs, 59 from the pregnant group and all dogs (n=19) from the non-pregnant group. Two samples were lost on the way to the laboratory. For 13 samples, results could not be interpreted adequately due to bad quality of the sample when it arrived at the laboratory, e.g., hemolytic serum, and were therefore excluded from the results. Of the remaining 65 dogs, 58 showed laboratory parameters within the reference range, or less than 2.5% above/below reference range, or unspecific changes of single parameters that were not regarded as clinically relevant. In seven dogs, blood parameters might be indicative of underlying disease (Table 2). Two dogs (pregnant) showed non-physiological changes in hematological parameters. One dog showed erythrocytosis with concomitant elevation of hemoglobin, and hematocrit. The other dog showed an unphysiological left shift in the differential blood count. Two dogs (pregnant: n=1, non-pregnant n=1) were classified as hypothyroid due to T4 measurements below the reference range with a concomitant increase in thyroid-stimulating hormone (TSH). Three dogs showed changes in serum biochemistry. None of the dogs showed clinical signs of disease.

**Table 2:** Results of hematology and blood chemistry indicative of underlying disease. Only parameters showing aberrations were included in the table. The remaining parameters were within reference range or less than 2.5% above/below reference range.

Parameter [unit]	reference range	Pregnant					Non-pregnant	
		**Dog 1**	**Dog 2**	**Dog 3**	**Dog 4**	**Dog 5**	**Dog 6**	**Dog 7**
Erythrocytes [T/L]	5.9-8.5	9.57						
Band neutrophils [/µL]	0		912					
Total T4 [nmol/L]	19.3-57.9			15.19			9.31	
TSH [ng/mL]	0.03-0.4			0.79			0.53	
α-amylase [U/L]	311-1142				4013			
DGGR-lipase [U/L]	<127				1827			466
Albumin [g/L]	28.1-39.4							25.4
Cholesterol [mmol/L]	4.3-10.5							16.29
Triglycerides [mmol/L]	0.4–2.8							3.53
Alkaline phosphatase [U/L]	<128							444
Glutamate-dehydrogenase [U/L]	<10.5							23.6
Creatinine [µmol/L]	<141					147.6		
Blood urea nitrogen [mmol/L]	3.2-11.5					11.66		

### NGS Analysis

#### Phylum level.

At phylum level, species belonging to 32 different phyla were found. The most common phyla were Bacteroidetes, Firmicutes, Proteobacteria, Actinobacteria, Fusobacteria, Tenericutes, Chloroflexi, Spirochaetes, and Synergistes (Table 3).

**Table 3:** Comparison of the most common phyla found in next generation sequencing (NGS) analysis of vaginal samples from pregnant (n=61) and non-pregnant (n=19) healthy breeding bitches, in decreasing relative abundance. Results represent the percentage of samples in which the respective phyla were identified.

Phylum	All animals	Pregnant	Non-pregnant
**Bacteriodetes**	100%	100%	100%
**Firmicutes**	100%	100%	100%
**Proteobacteria**	100%	100%	100%
**Actinobacteria**	96%	95%	100%
**Fusobacteria**	96%	98%	89%
**Tenericutes**	74%	70%	84%
**Chloroflexi**	70%	69%	74%
**Spirochaetes**	36%	34%	42%
**Synergistes**	19%	16%	26%

Bacteroidetes, Firmicutes, and Proteobacteria were found in every sample. Actinobacteria, Tenericutes, Chloroflexi, Spirochaetes, and Synergistes were less abundant in the pregnant group, whereas Fusobacteria were more abundant in the pregnant group.

#### Genus Level.

A total of 595 genera were found within the samples. *Bacillus, Bacteroides,* and *Escherichia/Shigella were* found in every sample (Table 4). Genera commonly known from bacteriological culture examinations of canine vaginal swabs were also common findings using NGS: *Haemophilus, Streptococcus*, and *Pasteurella* were identified in 95%, 91%, and 76% of the samples, respectively.

**Table 4:** Comparison of the most common genera found in next-generation sequencing (NGS) analysis of vaginal samples from pregnant (n=61) and non-pregnant (n=19) healthy breeding bitches, in decreasing relative abundance. Results represent the percentage of samples in which the respective genera were identified.

Genus	All animals	Pregnant	Non-pregnant
**Bacillus**	100%	100%	100%
**Bacteroides**	100%	100%	100%
**Escherichia/Shigella**	100%	100%	100%
**Paenibacillus**	99%	98%	100%
**Fusobacterium**	96%	98%	89%
**Prevotella**	96%	97%	95%
**Haemophilus**	95%	93%	100%
**Pontibacillus**	95%	95%	95%
**Porphyromonas**	95%	93%	100%
**Clostridium sensu stricto**	94%	95%	89%
**Parabacteroides**	94%	93%	95%
**Capnocytophaga**	93%	92%	95%
**Streptococcus**	91%	93%	84%
**Desulfosporomusa**	88%	87%	89%
**Geothrix**	88%	89%	84%

#### Mycoplasma.

**Fig 1:**
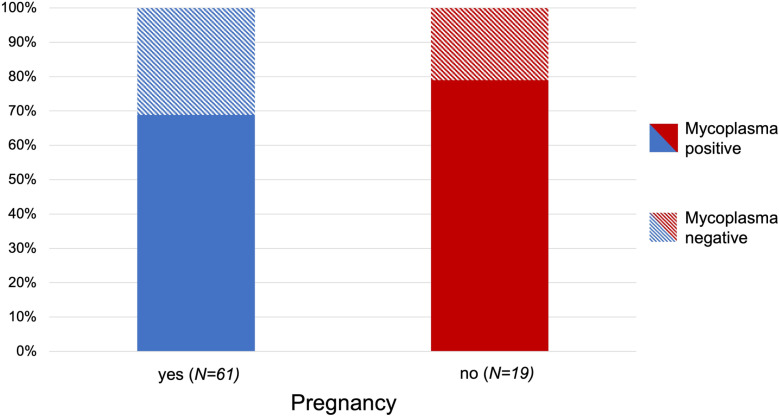
Prevalence of *Mycoplasma* in the vagina of pregnant (n **=61) and non-pregnant (n=19) healthy breeding bitches.** The samples were examined using Next-Generation Sequencing. Statistical analysis revealed no significance between groups (*p*=0.5634).

**Fig 2:**
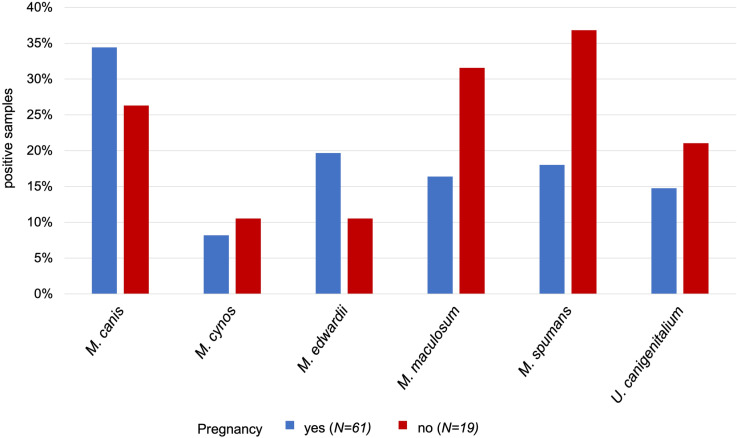
Comparison of clinically relevant and common *Mycoplasma* and *Ureaplasma* in the vagina of pregnant (n **=61) and non-pregnant (n=19) healthy breeding bitches.** The samples were examined using Next-Generation Sequencing. Statistical analysis revealed no significant differences between the groups indicating that pregnancy outcome was not influenced in the examined population by proof of specific *Mycoplasma* and *Ureaplasma*.

Prevalence of *Mycoplasma* was analyzed at species level. Both groups showed a high prevalence, with 69% (n= 42) in the pregnant group and 79% (n=15) in the non-pregnant group. The higher rate in the non-pregnant group was not statistically significant (*p*=0.5634). Results are shown in Figs 1 and 2 and Table 5.

**Table 5:** Total numbers and percentage of pregnant (n=61) and non-pregnant (n=19) healthy breeding bitches positive or negative for selected *Mycoplasma* and *Ureaplasma* species found in Next Generation Sequencing. Statistical analysis with Fisher’s Exact Test did not reveal significance. *P* values are given.

	Pregnant		Non-pregnant		*p*
	positive	negative	positive	negative	
*Mycoplasma canis*	21 (34%)	40 (66%)	5 (26%)	14 (74%)	0.5852
*Mycoplasma cynos*	5 (8%)	57 (92%)	2 (11%)	16 (89%)	0.6517
*Mycoplasma edwardii*	12 (20%)	49 (80%)	2 (11%)	17 (89%)	0.4996
*Mycoplasma maculosum*	10 (16%)	51 (84%)	6 (32%)	13 (68%)	0.1904
*Mycoplasma spumans*	11 (18%)	50 (82%)	7 (37%)	12 (63%)	0.1163
*Ureaplasma canigenitalium*	9 (15%)	52 (85%)	4 (21%)	15 (79%)	0.4959

#### Alpha-diversity and differential abundance analysis.

**Fig 3:**
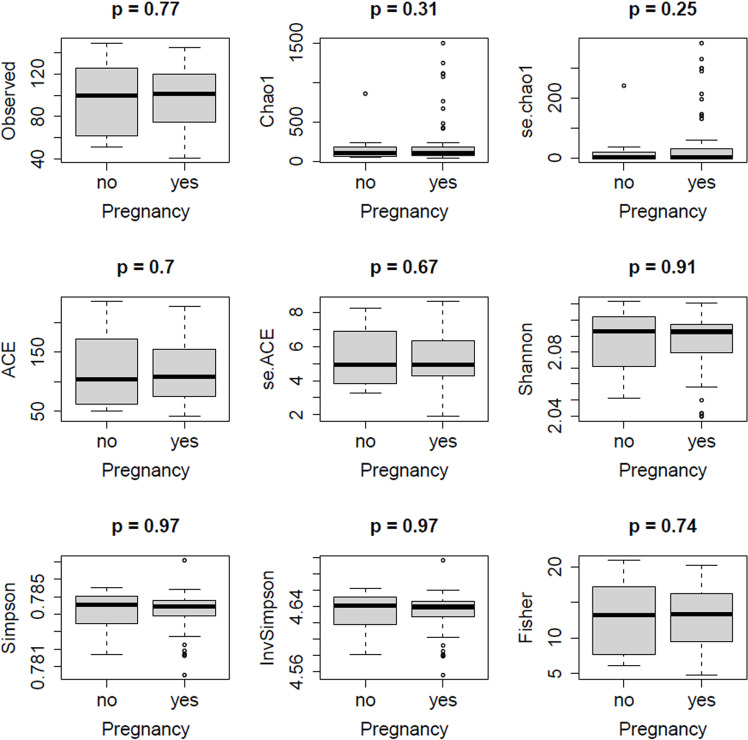
Comparison of **α-diversity within the vaginal microbiome of pregnant (*N*=61) and non-pregnant (*N*=19) healthy breeding bitches by different indices (Observed, Chao1, ACE, Shannon, Simpson, Fisher).** None of the indices provided evidence of differences between both groups.

Comparison of α-diversity by different standard measures did not reveal significant differences between the groups of pregnant and non-pregnant individuals. Calculated species richness scores and statistical analysis results are shown in Fig 3.

**Fig 4:**
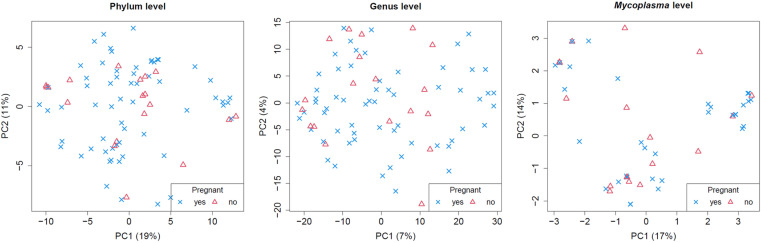
Principal Component Analysis on the level of genera, phyla, and *Mycoplasma* in vaginal samples of healthy breeding bitches. All samples were examined by Next-Generation Sequencing. Individual data are presented, differentiating healthy breeding bitches getting pregnant (*N*=61) or not (*N*=19). No obvious clustering according to pregnancy is visible.

**Fig 5:**
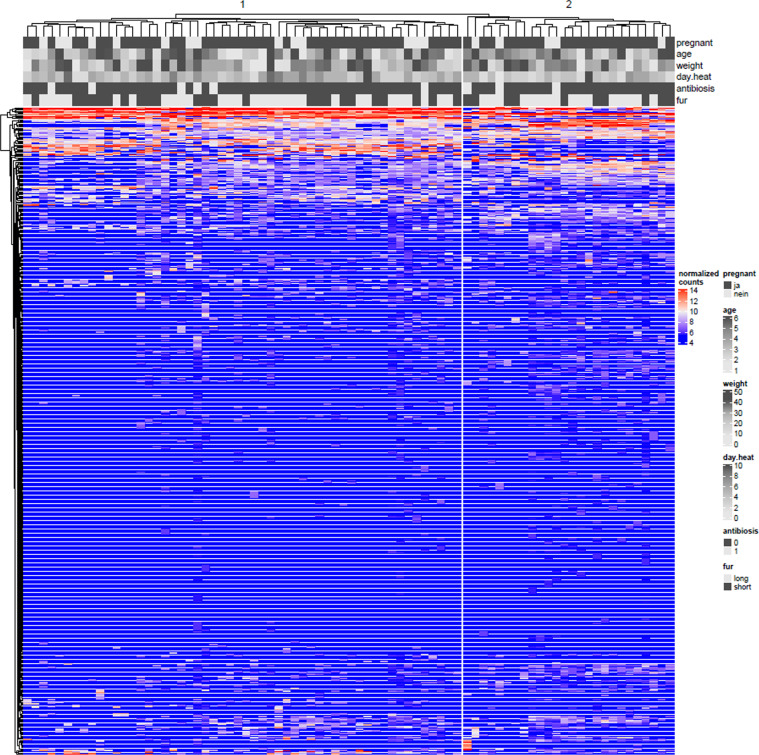
Clustered heatmap of vaginal samples from healthy breeding bitches. The genera are represented in rows, and animals getting pregnant (*N*=61) or not (*N*=19) are represented in columns. All samples were examined using Next-Generation Sequencing. No specific clustering according to pregnancy or other animal characteristics is detected.

Explorative and differential microbiome analysis were conducted at phylum level, genus level, and for *Mycoplasma* at species level. Neither principal component plots (Fig 4), nor clustered heatmaps (Fig 5) provided evidence of differences between pregnant and non-pregnant animals, despite there were two main clusters of samples that, however, could not be related to animal characteristics. Heatmaps on the phylum level and the *Mycoplasma* level are provided as Supplementary Material (S1 Fig **and** S2 Fig).

**Fig 6:**
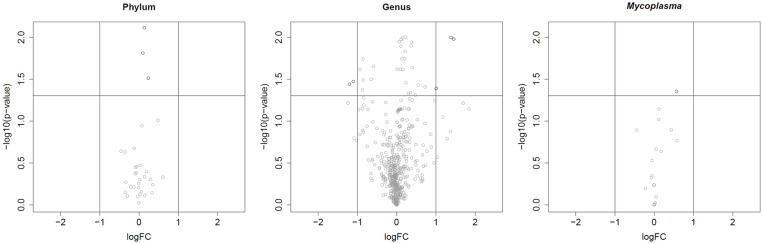
Volcano plot with the –log10 transformed unadjusted p-values versus the log fold change from the differential abundance analysis on the different taxonomic levels found in vaginal samples of healthy breeding bitches analyzed by Next Generation Sequencing. The horizontal line marks the significance level, i.e., –log10(0.05). While a few features are significant according to the raw *P*-value, significance is lost after *P*-value adjustment.

Differential abundance analysis on the different taxonomic levels revealed no significant differences after FDR-adjustment of *P*-values. A second model with age and antimicrobial treatment as potentially confounding variables did not yield significant effects by these two variables. The number of selected features according to different selection thresholds are provided in Table 6, results are also visualized in volcano plots (Fig 6). Full results of the differential abundance analysis are provided as Supplementary Material (S1-S3 Tables).

**Table 6:** Total numbers of selected features (phyla, genera or mycoplasma) with differential abundance between pregnant (*N*=61) and non-pregnant (*N*=19) healthy breeding bitches, according to different selection criteria. With the strongest criterion, the FDR^1^-adjusted p-value, no significant features were selected.

	# *P* < 0.05	# pFDR^1^ < 0.05	# |logFC^2^| > 1	# *P* < 0.05 & |logFC| > 1
**Phylum**	3	0	0	0
**Genus**	31	0	14	5
**Mycoplasma spp.**	1	0	0	0

^1^FDR: false discovery rate

^2^logFC: logarithmic fold change

## Discussion

Female fertility in dogs can be influenced by various factors, such as age [48–50], male semen quality [51], and most importantly inappropriate timing of the mating/insemination [31,52,53]. Similarly, the influence of the vaginal microbiota on reproductive health and fertility is controversial among veterinary specialists and breeders. It is well known that some bacteria might be causative to reproductive disease [29], but most species, with the exception of *Brucella canis*, are considered opportunistic pathogens [30,31]. Despite its limitations, such as the heterogeneity of the dog population (age, breed, parity), the lack of proof of ovulation for some animals (due to the owner’s decision), possible underlying disease in others and, most strikingly, a lack of follow-up samples after prescription of antimicrobials, this study is the first to examine a possible influence of the vaginal microbiome on fertility in bitches.

Next-Generation Sequencing analysis revealed a large variety of aerobic and anaerobic bacteria in the canine vaginal tract. Many genera commonly known from culture-based studies, such as *Escherichia/Shigella, Streptococcus, Haemophilus*, and *Pasteurella* [5,10,54], were detected, but in larger proportions, indicating that culture-based studies underestimate the prevalence of bacteria. In this context, Theron et al. (2000) postulated that < 10% of all bacteria present in a sample are identified by conventional culture-based techniques [55]. *Escherichia/Shigella* was found in every sample. A recent study on bull semen microbiota [56] had similar findings and proposed that this might be due to a contamination during sampling/sample processing. In this study, precautions to avoid contamination during sampling were taken, e.g., dry-cleaning of the vulva and the use of a sterile tube speculum. Furthermore, controls were used during sample processing to identify possible contaminants. Nonetheless, it cannot be completely ruled out that *Escherichia/Shigella* might be a contamination. However, *Escherichia coli* is among the most common bacteria found in the canine vaginal tract in previous studies [5,10]. In the context of canine pyometra, it is often postulated that *E. coli* in the canine vagina might ascend from the gastrointestinal tract [57]. This hypothesis is supported by studies investigating the relationship between vaginal and gastrointestinal strains of *E. coli* [58,59]. Although this was not investigated in vaginal samples of healthy bitches, we hypothesize that the microbiota of the gastrointestinal tract might have an influence on the vaginal microbiota in all bitches due to the anatomic proximity of the anus and vulva.

Previous culture-based studies mostly used aerobic conditions, hence knowledge on anaerobic bacteria in the canine vaginal tract is scarce [4,7]. However, the frequent identification of anaerobic genera, such as *Bacteroides, Fusobacterium, Prevotella, Porphyromonas* and *Clostridium sensu stricto*, clearly supports that the canine vagina also harbors a physiological anaerobic flora.

Even though to our knowledge this is the first study correlating the vaginal microbiome with fertility and pregnancy outcome, two previous studies investigated the vaginal microbiome of dogs[25,26]. Whereas the dominant phyla from these studies were comparable to our results, Lyman et al. found a different result on genus level, with *Hydrotalea, Ralstonia*, and *Mycoplasma* being the most common genera. Differences between previous and our study might be attributable to differences in cycle stages (Lyman et al. examined 5 dogs for each cycle stage, prepubertal, proestrus, estrus, diestrus, anestrus, whereas we only used bitches (N=80) in proestrus), age (Lyman et al. used “young” bitches without further specification, we used adult bitches), or previous matings (Parity is not given in Lyman et al., whereas our population is/mixed with mostly primi-/pluriparous and some nulliparous bitches). Similar to earlier studies, heterogeneity in terms of breed and age, but also possible other factors is a limiting factor of our study, though our study population clearly represents the dog population presented for breeding management in a clinical setting. An influence of the cycle stage was described in culture-based approaches [5,8,10], and an NGS study revealed an impact of age in dogs [26]. In humans, an influence of sexual intercourse on the vaginal microbiome was shown [60]. Previous matings and therefore parity might also have an influence in dogs; however, this has yet to be proven.

*Mycoplasma* are possibly the most feared bacteria among breeders, although they were previously found in the vaginal tract of both healthy and diseased bitches [10,36,61]. In our investigation, the prevalence of *Mycoplasma* was high in both groups and did not differ statistically. Interestingly, *Mycoplasma canis*, which was postulated to be associated with reproductive disease in bitches [39], had a higher prevalence in the pregnant group. No significant statistical difference between pregnant and non-pregnant animals was found for any of the *Mycoplasma* in this study, neither was evidence found within the bioinformatic analysis. This strongly suggests that a bitch testing positive for *Mycoplasma* should not receive antimicrobial treatment unless this is justified by the combination of individual history, clinical signs, and findings in gynecological examination. This finding is especially noteworthy, as long courses of tetracyclines or fluoroquinolones are the therapy of choice [37] and (non-indicated) treatment bears the potential of facilitating antimicrobial resistance against critically important antimicrobials. Sharing our findings with veterinarians and breeders is therefore crucial in terms of a one-health approach. However, possible statistical errors due to the small sample size must be considered and investigations of the different *Mycoplasma* in larger cohorts are needed to further support our hypothesis.

Bioinformatic analysis of the present data did not reveal any evidence of significant difference in the composition of the vaginal microbiome between pregnant and non-pregnant animals. This might suggest that the often postulated, but scarcely investigated influence of the microbiome on fertility might be overestimated. Although detecting no differences is not final proof of a missing correlation between the vaginal microbiome and canine fertility and larger sample sizes, due to larger statistical power might have even revealed significance, the potential effects appear not to have a biological relevance due to the rather small fold changes observed.

Nevertheless, it is undeniable that gynecological disease might be associated with bacteriological culture of specific bacteria, such as *E. coli* [29–32]. The present data emphasize once more the opportunistic character of these organisms, as the genus *Escherichia/Shigella* was detected in all samples in this study. None of the genera could be identified as a marker for suspected subfertility/infertility. Moreover, underlying causes for the opportunistically pathogenic action of the vaginal microbiota should be investigated further. These might be over-growth in the absence of competing commensal bacteria [9], or the local immune response and metabolic milieu. The latter two are described in humans as part of a complex interplay between the host and vaginal microbiome, mutually influencing each other [62]. The immune response also plays a role in the establishment of canine pregnancy within the uterine tissues, with a shift to rather anti-inflammatory conditions [63]. To the best of our knowledge, the role of immune modulation in the vagina of dogs has not been studied to date. Undoubtedly, the regulating mechanisms and interplay between the microbiome and the host are yet to be understood in dogs. Moreover, bacterial vaginosis, the most important vaginal dysbiosis in women, is correlated with impaired fertility and adverse reproductive and gynecological outcomes, such as preterm birth and endometritis [62,64,65]. Infertile women have distinct microbiomes compared to fertile women [62]. However, being dominated by *Lactobacillus* [65], the human vaginal microbiome substantially differs from the canine vaginal microbiome [25,26]. *Lactobacillus* were identified in our samples but were not dominant within the canine vaginal microbiome. This is in agreement with Lyman et al. [25] who found a low abundance of lactobacilli (0.03% of the total population) and postulated this might be attributable to the almost neutral to alkaline pH in the canine vaginal tract [25]. Despite the differences, future research should focus on the characterization of canine vaginal dysbiosis and its impact on gynecological health as well as fertility. Nevertheless, indices estimating the diversity within the microbiome did not reveal significant differences between pregnant and non-pregnant animals within this study population.

In terms of possible dysbiosis, it is noteworthy that bitches receiving antimicrobial treatment had significantly lower pregnancy rates. Next-Generation Sequencing samples were obtained before the start of treatment, so NGS results were not influenced, and treatment was therefore not included as a cofounding factor in the bioinformatics analysis. It was out of the scope of this study to take follow-up samples after treatment to investigate the impact of antimicrobial treatment on the canine vaginal microbiome. However, as we did not follow this up, it remains to be clarified whether pregnancy outcome was related to microbiota findings, antimicrobial treatment, or possibly unknown underlying alterations of, for example, the endometrium. Treatment decision was based on a history of impaired fertility and/or pure culture of potentially pathogenic bacteria identified by conventional culture-based technique (sampled at the day of NGS sampling). This needs to be taken into consideration when interpreting lower pregnancy rates with antimicrobial treatment. This finding might as well be attributable to the aforementioned characteristics of the bitches within the group that received antimicrobials, namely especially a history of infertility. Therefore, no conclusion can be drawn from this finding and further investigations on the effect of antimicrobial treatment on the vaginal microbiome, and a possible influence on fertility, are needed to verify or reject this hypothesis. In this context, it is noteworthy that one of the treated bitches not conceiving was diagnosed with suspected hypothyroidism, thus clearly supporting the need for a detailed workup of “infertile” bitches, including blood work. Despite antimicrobial treatment based on culture-results being a common practice, it is known that culture-based methods detect only up to 10% of the bacteria present in a microbial ecosystem [55]. Corresponding data from the NGS analysis strongly suggests that pure cultures do not exist in the canine vagina, as numerous genera were detected by NGS in every sample. Antimicrobial treatment influences all susceptible bacteria within the treated host and might therefore also deplete commensal (and possibly beneficial bacteria) and cause disbalance within the vaginal microbiome, allowing resistant and possibly pathogenic bacteria to proliferate in the absence of competition for nutrients and cell adhesion [9]. It is widely accepted that disturbances of the intestinal microbiome are a consequence of antimicrobial exposure in humans [66] as well as in dogs [67]. Although no data on the influence of antimicrobial treatment on the canine vaginal microbiome are available, a (possibly negative) alteration of the vaginal microbiome should be taken into consideration. The decision on whether to use an antimicrobial should therefore not be based solely on the results of conventional microbiological tests (including detection of pure cultures), but rather rely on clinical features like signs of inflammation, as, e.g., purulent vaginal discharge and/or significant numbers of neutrophils in the vaginal cytology smear. Next Generation Sequencing could possibly provide an addition to clinical diagnostics in the future, especially with novel NGS approaches offering fast turnaround time, high accuracy and affordable costs per sample. However, this is currently not feasible due to the lack of a knowledge on the physiological and pathological canine vaginal microbiome. Further studies, possibly including healthy as well as diseased animals, are needed to obtain information on potential markers of a pathological microbiome and define references for the clinician. Diversity indices might be a promising tool as “reference parameters”.

Another well-known factor negatively influencing female fertility is age. Reduced fertility as indicated by reduced litter size [48–50], or even unsuccessful matings [50] have been repeatedly described to be associated with increased age and non-pregnant animals were significantly older in this study, too (*P*<0.01). Although age-dependent differences in the canine vaginal microbiome were recently described by Hu et al. [26], we did not find such differences; however, prepubertal and senior animals were not included in our study.

Underlying disease, other than reproductive-related disease, also needs to be considered as a possible reason for infertility/subfertility. To identify possible causes, detailed blood analysis was performed in all obviously clinically healthy dogs revealing deviations in hematology (*N*=2), serum biochemistry (*N*=3), and thyroid function (*N*=2) in seven dogs. Identified hematology abnormalities were erythrocytosis without signs of hemoconcentration (no increased serum or plasma protein concentration) (*N*=1), and neutrophilia with left shift in the differential blood count (*N*=1). Absolute erythrocytosis can be rarely primary (polycythemia vera or other erythroid neoplasia), but rather secondary due to an increase in erythropoietin concentration related to hypoxia (chronic cardiopulmonary disease, right-to-left cardiovascular shunts), or due to different neoplasms or renal disorders that produce erythropoietin [68]. Furthermore, epinephrine release can lead to splenic contraction with subsequent physiological erythrocytosis. As the bitch was otherwise healthy and no follow-up diagnostic was conducted in this case, the etiology of erythrocytosis remains unknown. Nevertheless, pregnancy in this dog was normal and she whelped nine healthy puppies without any complications. Interestingly, she was hospitalized due to severe pneumonia later in the weaning phase, possibly pointing to an underlying undiagnosed and unobserved chronic pulmonary disease. The observed neutrophilia with a left-shift in the other bitch can be indicative of acute inflammatory disease [69]. However, the dog was otherwise healthy and showed no other changes in the blood analysis. As the mating was successful and the bitch whelped six healthy puppies without any complications, the finding seems not to be of relevance in this case. Regarding changes in serum biochemistry analysis (*N*=3), elevated activities of pancreatic enzymes (*N*=2; pregnant/non-pregnant: 1/1) possibly indicative of exocrine pancreatic disease [70] and mild azotemia (*N*=1, pregnant) were found. Azotemia is either classified as prerenal (in case of severe dehydration, cardiac failure), renal (in case of renal disease), or postrenal (in case of urinary tract obstruction, uroabdomen) [71]. Although the dog showed no signs of hemoconcentration and was clinically healthy, further diagnostic tests (urinalysis, SDMA analysis, abdominal ultrasound) were advised to the owner, who did not consent thereto. Whereas the impact of exocrine pancreatic disease and kidney disease on (canine) fertility is unknown, impaired fertility is one of the most common complications of chronic kidney disease (CKD) in humans [72]. Interestingly, hypothyroidism was diagnosed in two dogs (pregnant/non-pregnant: 1/1), supporting previous research that hypothyroidism might interfere with the reproductive performance of dogs. However, different from women [73], evidence is contradictory [74–77]. Due to the small numbers of bitches with abnormalities in the blood analysis, our data do not allow for final conclusions to be drawn on the etiology of sub-/infertility in the specific cases nor on the impact of the findings on canine fertility in general. It was not within the scope of the present study to assess the impact of possible underlying disease on fertility in general, however, this must be discussed as a influencing factor.

Despite its novelty and clinical relevance, it is important to consider the limitations of the present study. As previously discussed, fertility is influenced by a multitude of factors. Not all of these could be controlled by the study design, as the recruited animals were all client-owned dogs. The most important factor is the correct timing of the mating. Proof of ovulation (by progesterone measurement) was assessed for most dogs. However, for some of the animals either no proof of ovulation was obtained, or no reliable information was available. Although statistical analysis revealed no significance, missing proof of ovulation and therefore a potential incorrect timing of the mating must be considered as a possible reason for a lack of pregnancy in the respective bitches. Another limitation is the different type of mating. Some of the bitches were artificially inseminated. Regarding the success rates, artificial insemination is considered inferior to natural mating [78,79], despite clinical experience might be different. Furthermore, whelping rates after artificial insemination using frozen-thawed semen are described to be significantly lower compared to fresh semen [80]. Frozen-thawed semen was used in three bitches of which two became pregnant. These animals were inseminated by a specialized veterinarian in reproduction using endoscopic transcervical insemination (TCI). Transcervical insemination is the technique with the highest success rate [78]. Ten bitches were inseminated with either fresh semen or chilled semen. Most of the inseminations using fresh semen/chilled semen were carried out by specialist veterinarians using TCI if possible. In some cases, the inseminations were carried out by others (e.g., due to the location of the stud dog) and no further information on the procedures could be obtained. Both the use of artificial insemination and the lack of a standardized procedure in all inseminated dogs need to be considered as a limitation. Lack of pregnancy in these animals must be interpreted with caution, as it might be related to the wrong timing or the type of mating. Nevertheless, statistical analysis did not reveal differences between the dogs becoming pregnant and those not becoming pregnant. Another factor being considered as possible limitation is heterogeneity in breeds. Due to the variable numbers of dogs belonging to one breed, ranging from one to maximum 13, statistical analysis whether the breed of the dogs had an influence on the composition of the vaginal microbiome was not justified. Nevertheless, a possible breed effect should be investigated in future studies.

While a more homogenous group of study animals would have been preferable in terms of study design, this rather heterogenous population of dogs perfectly represents the breeding dog population in a clinical setting. Follow-up studies should aim to better control these limitations by selecting a more homogenous group of study animals and using standardized protocols for the matings.

The results of our study show a large diversity within the vaginal microbiome of healthy breeding bitches. The canine vagina harbors different bacterial genera, including *Escherichia/Shigella* and *Mycoplasma*, independent of pregnancy outcome. Our results underline the richness of the vaginal microbiome as well as the opportunistic nature of the bacteria. Furthermore, they question the benefits of pre-mating culture-based bacteriological examinations in gynecologically healthy bitches because the cultures are often misinterpreted, leading to over-use of antimicrobials. The limitations, most importantly the heterogeneity of the bitches in the present study, need to be considered and the results must be interpreted taking this variation into account. Further investigations should avoid these factors. Although we cannot completely rule out other reasons for infertility, future studies should include follow-up NGS examinations in bitches treated with antibiotics and record fertility to gain insights into the role of canine vaginal mucosa immunity and potential dysbiosis after treatment. Moreover, a comparative investigation of the microbiome of reproductive-diseased and healthy bitches on a larger scale could provide reference data to define canine vaginal dysbiosis.

## Supporting information

S1 Fig Clustered heatmap with phyla represented in rows and samples represented in columns. No specific clustering according to pregnancy or other animal characteristics is detected. (TIF)

S2 Fig Clustered heatmap with *Mycoplasma* represented in rows and samples represented in columns. No specific clustering according to pregnancy or other animal characteristics is detected. (TIF)

S1 Table Differential abundance analysis on genus level The differential abundance analysis on genus level did not reveal significant differences after FDR-adjustment of p-values. (XLSX)

S2 Table Differential abundance analysis on phylum level The differential abundance analysis on phylum level did not reveal significant differences after FDR-adjustment of p-values. (XLSX)

S3 Table Differential abundance analysis of *Mycoplasma* The differential abundance analysis of *Mycoplasma* did not reveal significant differences after FDR-adjustment of p-values. (XLSX)
