# Supplementary figures and images for: The canine vaginal microbiome during heat and fertility in healthy breeding dogs
Source: PLoS One. 2025 Apr 28;20(4):e0321683. doi: 10.1371/journal.pone.0321683 (PMC12036845; doi:10.1371/journal.pone.0321683)

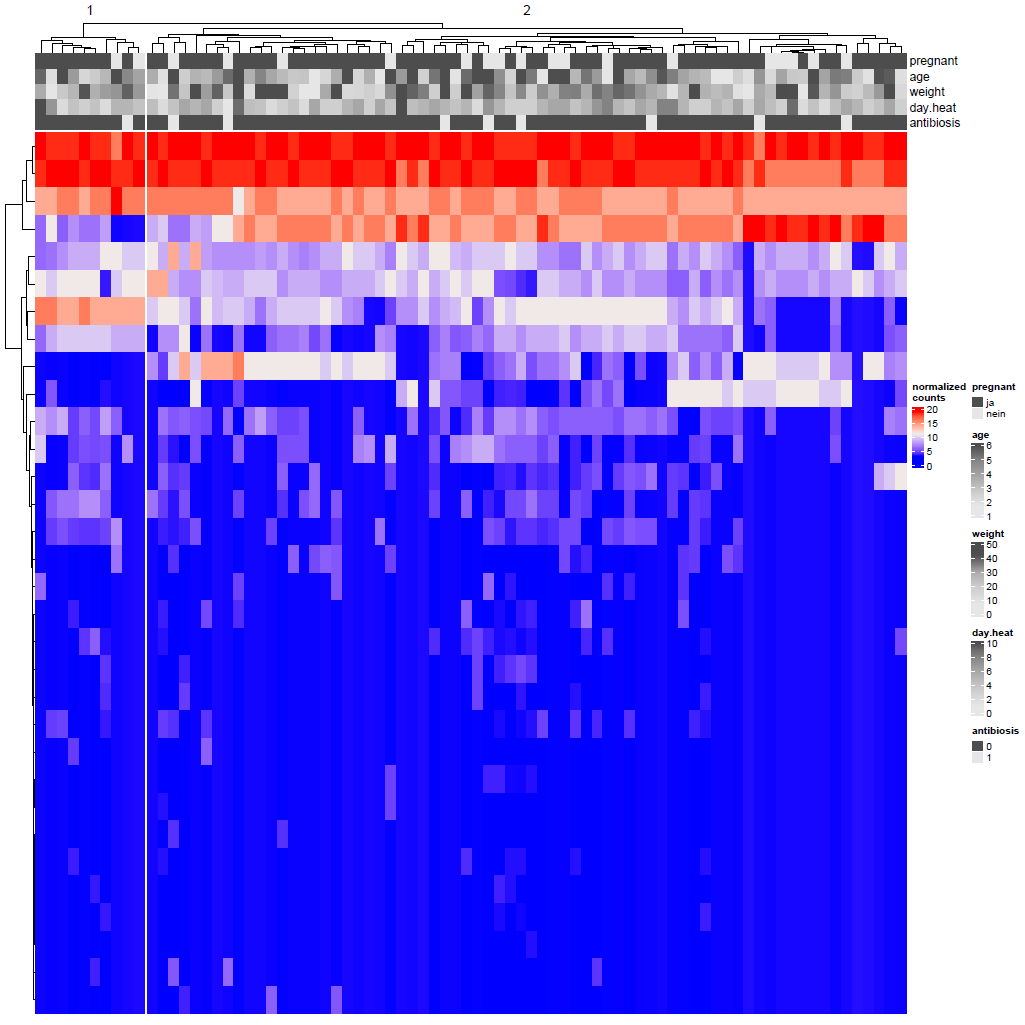

Supplement: S1 Fig — (TIF) [file pone.0321683.s001.tif]

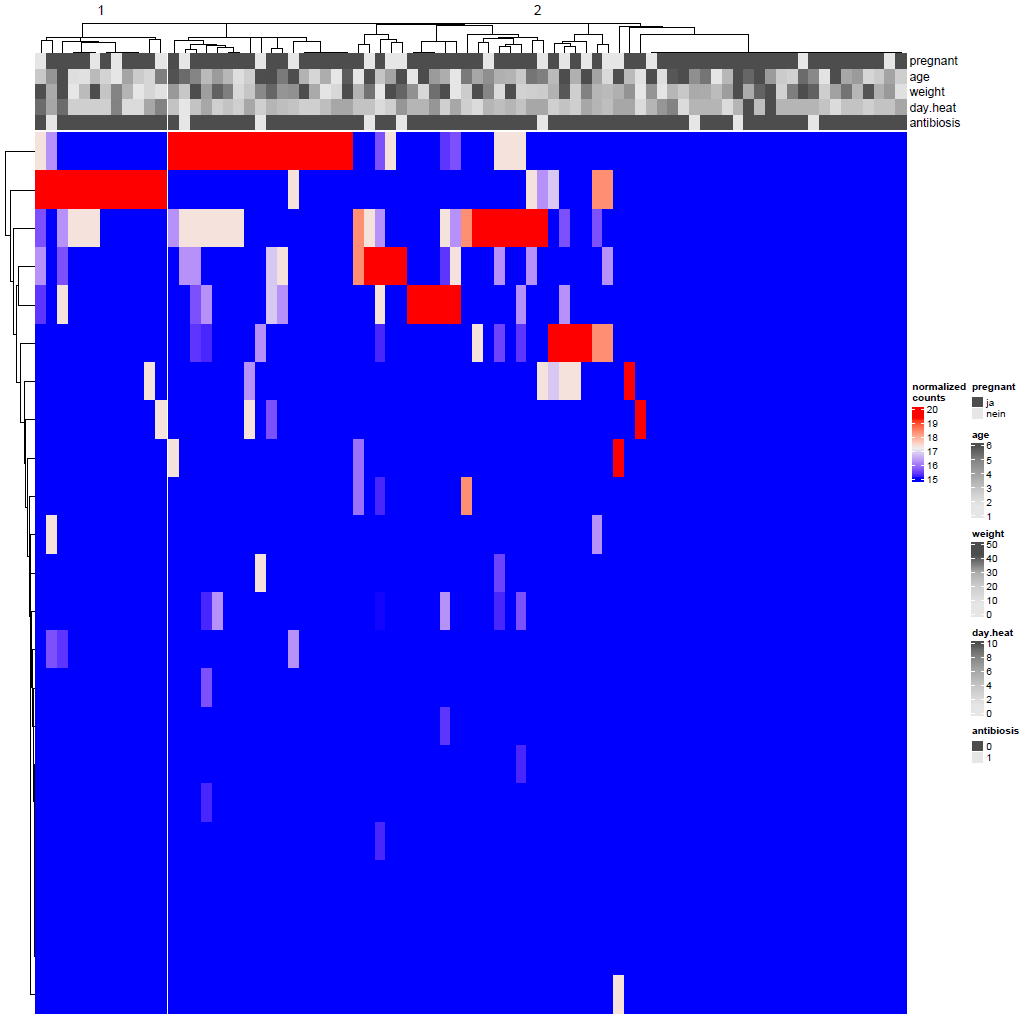

Supplement: S2 Fig — (TIF) [file pone.0321683.s002.tif]
